# Synthesis and Antimicrobial Activity of Some New 1,3,4-Thiadiazole Derivatives

**DOI:** 10.3390/molecules171214625

**Published:** 2012-12-10

**Authors:** Thoraya A. Farghaly, Magda A. Abdallah, Mohamed R. Abdel Aziz

**Affiliations:** Department of Chemistry, Faculty of Science, Cairo University, Giza 12613, Egypt

**Keywords:** antimicrobial activity, enaminone, 1,3,4-thiadiazole, hydrazonoyl halides

## Abstract

New series of 1,3,4-thiadiazoles have been prepared *via* reaction of 1,3,4-thiadiazolenaminones **1** with *N*-phenyl 2-oxopropanehydrazonoyl chloride (**2**) in dioxane in the presence of triethylamine. Also, some new heterocycles incorporating 1,3,4-thiadiazole ring were obtained by reaction of 1,3,4-thiadiazolenaminones **1** with nitrogen-nucleophiles like hydrazine hydrate, 3-amino-1,2,4-triazole and 2-aminobenzimidazole. The structure of the new products was established based on elemental and spectral analysis. The relation between the structure of the products and their activity towards some microorganisms was studied and promising results were obtained.

## 1. Introduction

Recently, the chemistry of enaminones has received considerable attention due to their utility as building blocks in heterocyclic synthesis [1,2,3,4,5]. On the other hand, 1,3,4-thiadiazole derivatives have attracted considerable interest owing to their wide spectrum of biological activity, including anti-microbial, anti-tuberculosis, anticonvulsant, anti-inflammatory, and antiulcer properties [6,7,8,9,10]. Recently, we published the antimicrobial activity results of a series of *N*-[3-aryl-5-(3-dimethylamino-acryloyl)-3*H*-[1,3,4]-thiadiazol-2-ylidene]-benzamides, which showed promising activity [11]. Based on these findings, and in continuation of our interest in synthesis of bioactive compounds [12,13,14,15,16], we have now prepared a new series of 1,3,4-thiadiazoles *via* reaction of *N*-[3-aryl-5-(3-dimethylamino- acryloyl)-3*H*-[1,3,4]-thiadiazol-2-ylidene]-benzamides with 1,3-dipoles and some nitrogen nucleophiles to investigate the antimicrobial activity of the products and study their structure activity relationship (SAR) towards some microorganisms.

## 2. Results and Discussion

**Scheme 1:**
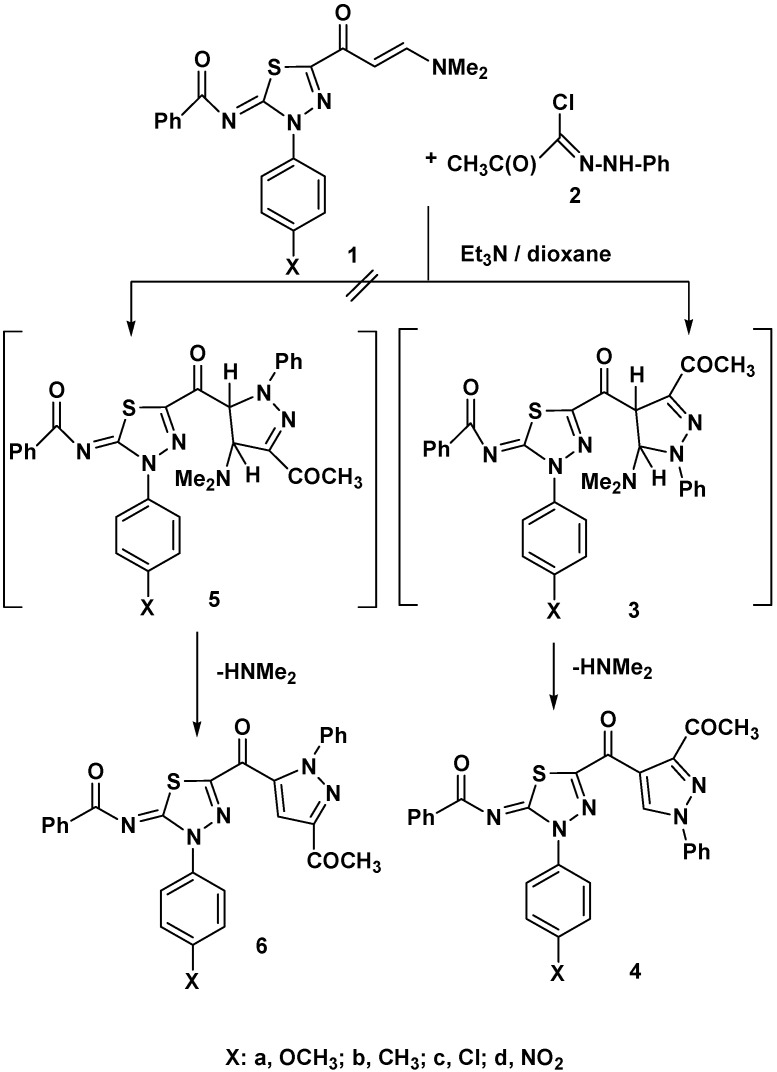
Reaction of enaminones **1a**–**d** with hydrazonoyl chloride **2**.

Recently, 1,3,4-thiadiazole-enaminones **1a**–**d** were prepared in our laboratory *via* reaction of 5-acetyl-3-aryl-2-benzoylimino-1,3,4-thiadiazoles with dimethylformamide-dimethylacetal (DMF-DMA) under reflux in dry toluene [11]. The reactions of these enaminones as dipolarophiles with 1,3-dipoles were studied next. Thus, reaction of **1a**–**d** with hydrazonoyl chloride **2** in refluxing dioxane in the presence of triethylamine afforded, in each case, one isolable product, as evidenced by TLC analysis. The structures of the isolated products were identified, based on their elemental analyses and spectral (IR, ^1^H-NMR, ^13^C-NMR and MS) data, as the respective 3-aryl-2-benzoylimino-5-(1-phenyl-3-acetyl-pyrazol-4-yl-carbonyl)-1,3,4-thiadiazoles **4a**–**d** (Scheme 1). 

For example, the IR spectra of products **4** revealed in each case, three C=O absorption bands in the 1694–1680, 1642–1636 and 1617–1604 cm^−1^ region. Also, their ^1^H-NMR spectra exhibited in each case characteristic singlet signals at 2.44–2.38 and 9.34–9.31 ppm assigned to the protons of the acetyl group and pyrazole-CH, respectively. The ^13^C-NMR of compounds **4a**–**d** revealed three signals at 176–172, 190–189 and 206–196 for the carbons of the three carbonyl groups. The mechanism of formation of products **4** is depicted in Scheme 1. The suggested pathway is consistent with all reports published about the reaction of hydrazonoyl halides with enaminones which indicate that this reaction leads to the formation of 5-unsubstituted pyrazole derivatives [11,17,18] and not the regioisomeric 4-unsubstituted pyrazoles **6**.

**Scheme 2:**
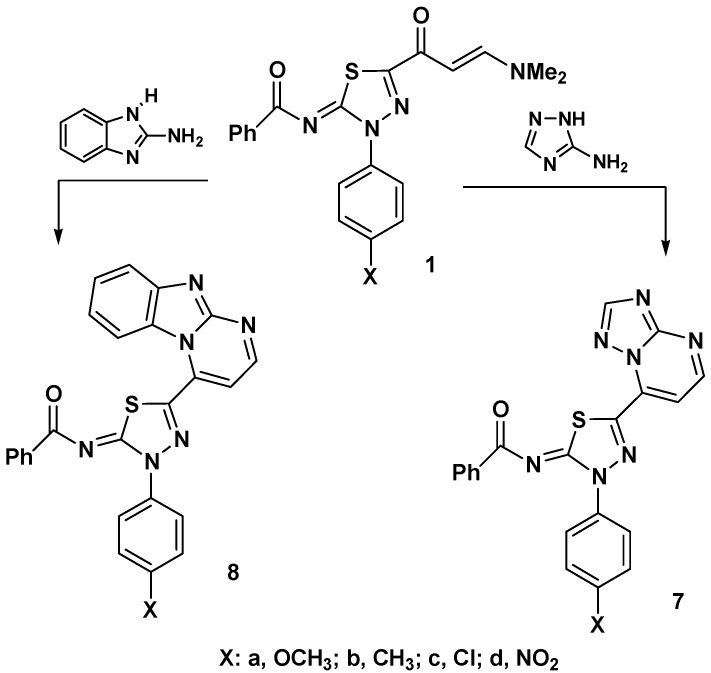
Reaction of enaminones **1a**–**d** with heterocyclic amines.

The reactivity of thiadiazole-enaminones **1a**–**d** with some nitrogen-nucleophiles was studied next. Thus, reaction of **1a**–**d** with 3-amino-1,2,4-triazole in acetic acid under reflux led to formation of 1,2,4-triazolo[1,5-*a*]pyrimidine derivatives **7** (Scheme 2). Similarly, reaction of **1a**–**d** with 2-aminobenzimidazole under the same reaction conditions afforded the respective benzimidazo[1,2-*a*]pyrimidines **8** (Scheme 2). 

The pathway of formation of products **7** and **8** involves Michael addition of the exocyclic amino group of the heteroamines to the enaminone double bond of **1**, followed by in *situ* tandem elimination of dimethylamine and dehydrative cyclization. The structure of the products **7** and **8** was confirmed based on elemental and spectral data (see Experimental). For example, the IR spectra of products **7** and **8** revealed in each case the absence of the carbonyl absorption band due to the enaminone residue in compounds **1**. Also, the ^1^H-NMR spectrum of each of the products **7** and **8** displayed two doublets in the regions 9.71–8.14 and 8.56–8.12 ppm with *J* values near 5 Hz that are assignable to the two vicinal protons in the pyrimidine moieties [19,20,21]. 

**Scheme 3:**
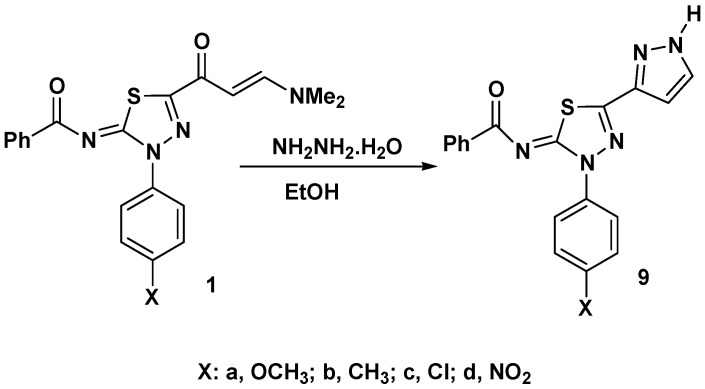
Reaction of enaminones **1a**–**d** with hydrazine hydrate.

On the other hand, reaction of enaminones **1a**–**d** with hydrazine hydrate in ethanol under reflux led to formation of the thiadiazole-pyrazole linked products **9** (Scheme 3). The structure of the latter products was established using spectroscopic and elemental analysis methods. For example, the IR spectra of products **9** revealed in each case only one carbonyl band near 1610 cm^−1^ attributed to the benzoylimino group. In addition, the ^1^H-NMR spectra of products **9** exhibited a singlet signal at δ 9.0–10.34 ppm due to the NH proton of pyrazole ring. 

### 2.1. Biological Screening

#### 2.1.1. Antimicrobial Activity

In *vitro* antimicrobial screening of compounds **4**, **7**, **8** and **9** prepared in this study was carried out using cultures of four fungal strains, including *Aspergillus fumigatus* (RCMB 002003) (*AF*), *P. italicum* (RCMB 005003, *PI*), *Geotrichum candidum* (RB052006, *GC*), and *Candida albicans* (RCMB 005002, *CA*) as well as four bacteria species, namely, Gram positive bacteria, *Staphylococcus aureus* (RCMB 000106, *SA*) and *Bacillus subtilis* (RCMB 000107, *BS*), Gram negative bacteria, *Pseudomonas aeruginosa* (RCMB 000102, *PA*) and *Escherichia coli* (RCMB 000103, *EC*). Amphotericin B as an antifungal agent, ampicillin as an antibacterial agent for Gram positive bacteria and gentamicin as an antibacterial agent for Gram negative bacteria were used as references to evaluate the potency of the tested compounds under the same conditions. 

#### 2.1.2. Antimicrobial Activity Screening and Structure Activity Relationship

The results of antimicrobial activities for some of the newly synthesized compounds showed promising effects compared to control drugs (see Table 1). Compounds **4a** and **4b** have high potency as antifungals except for the fungus *Candida albicans* (CA). Replacing the substituent X in the phenyl group at position 3 of the 1,3,4-thiadiazole moiety in compounds **4** with electron withdrawing groups, e.g., X = Cl, NO_2_, as in **4c** and **4d**, leads to a decrease in the antifungal activity to zero (Table 1). The results also showed that compounds **4a** and **4b** have high potency towards Gram positive bacteria *SA* and *BS* and Gram negative bacteria *PA*. Their antibacterial activity is high compared with compounds **4c**,**d**, which is in agreement with what was mentioned before. On the other hand, compounds **7c** and **7d** have higher potency against all tested fungi except *CA* than compound **7a**. Compound **7b** has no activity towards any of the tested fungi. In addition, compounds **7a**, **7c** and **7d** have high activity against almost all bacteria used, while compound **7b** has no activity against any of the tested bacteria. This indicates that replacing X in the phenyl group at position-3 of 1,3,4-thiadiazole moiety in compounds **7** with an electron donating group decreased the activity of these compounds towards all tested microorganisms. In addition, the results depicted in Table 1 revealed the high potency of compounds **8** towards all tested microorganisms, except fungus *CA* and Gram negative bacteria *PA*. The order of decreasing reactivity towards tested fungi and bacteria is as follows: **8b** > **8c** > **8a**. Furthermore, the results of Table 1 indicated that compounds **9a** and **9b** have high potency towards all tested fungi except fungus *CA* and Gram negative bacteria *PA*. The activity of compounds **9a**,**b** can be attributed to the presence of pyrazole ring and the small size of the molecules. 

**Table 1:** Antimicrobial activity expressed as inhibition diameter zones in millimeter (mm) of compounds **4**, **7**, **8** and **9** against the pathological strains based on well diffusion as assay.

Compound No.	Fungi				Gram positive bacteria		Gram negative bacteria	
	*A. fumigatus*	*P. italicum*	*C. albicans*	*G. candidum*	*S. aureus*	*B. subtilis*	*P. aeruginosa*	*E. coli*
**4a**	15.6 (±0.19)	16.3 (±0.24)	N.A.	19.8 (±0.38)	17.9 (±0.27)	20.2 (±0.14)	N.A.	10.9 (±0.29)
**4b**	19.6 (±0.13)	20.3 (±0.22)	N.A.	22.4 (±0.14)	22.8 (±0.25)	25.8 (±0.31)	N.A.	17.6 ±0.25
**4c**	10.3 (±0.12)	N.A.	N.A.	N.A.	12.0 (±0.21)	12.3 (±024)	N.A.	8.4 (±0.12)
**4d**	N.A.	N.A.	N.A.	N.A.	13.6 (±0.17)	15.4 (±0.33)	N.A.	N.A.
**7a**	13.9 (±0.25)	16.7 (±0.19)	N.A.	19.8 (±0.35)	11.7 (±0.14)	14.6 (±0.67)	N.A.	N.A.
**7b**	N.A	N.A.	N.A.	N.A	N.A	N.A	N.A.	N.A.
**7c**	18.5 (±0.15)	19.2 (±0.11)	N.A.	20.9 (±0.26)	18.3 (±0.19)	19.2 (±0.21)	N.A.	12.2 (±0.13)
**7d**	14.8 (±0.13)	12.6 (±0.21)	N.A.	16.8 (±0.22)	15.0 (±0.18)	11.2 (±0.12)	N.A.	11.3 (±0.31)
**8a**	14.3 (±0.21)	15.2 (±0.23)	N.A.	11.7 (±0.15)	15.8 (±0.31)	18.6 (±0.21)	N.A.	N.A.
**8b**	20.4 (±0.19)	21.6 (±0.12)	N.A.	25.8 (±0.37)	23.9 (±0.27)	26.7 (±0.14)	N.A.	19.8 (±0.10)
**8c**	16.9 (±0.13)	17.8 (±0.24)	N.A.	21.4 (±0.17)	19.4 (±0.27)	20.4 (±0.14)	N.A.	10.6 (±0.31)
**9a**	20.3 (±0.31)	N.A.	N.A.	22.6 (±0.22)	23.7 (±0.31)	25.9 (±0.22)	N.A.	15.9 (±0.38)
**9b**	21.6 (±0.22)	20.8 (±0.12)	N.A.	26.2 (±025)	22.0 (±0.23)	21.4 (±012)	N.A.	18.9 (±0.26)
Amphotericin B	23.7 (±0.10)	21.9 (±0.12)	19.8 (±0.20)	28.7 (±0.22)	N.A.	N.A.	N.A.	N.A.
Ampicillin	N.A.	N.A.	N.A.	N.A.	27.4 (±0.18)	32.4 (±0.10)	N.A.	N.A.
Gentamicin	N.A.	N.A.	N.A.	N.A.	N.A.	N.A.	17.3 (±0.15)	22.3 (±0.18)

The experiment was carried out in triplicate and average zone of inhibition was calculated (100 µL was tested) (N.A. = no activity), data are expressed in the form of mean ± SD.

#### 2.1.3. Minimum Inhibitory Concentration (MIC)

The minimum inhibitory concentration (MIC) of the synthesized compounds against highly inhibited organisms is reported in Table 2. Compound **9b** revealed high a MIC value of 0.9 µg/mL against *Aspergillus fumigatus* (RCMB 002003), 0.08 µg/mL against *Geotrichum candidum* and 1.95 µg/mL against *Straphylococcus aureus.* Compounds **4c** and **9a** exhibited a low MIC value of 0.12 µg/mL against Gram positive bacteria (BS), while compound **8b** revealed a MIC of 7.81 µg/mL against *PI*.

**Table 2:** Minimum inhibitory concentration (µg/mL) against the pathological strains.

CompoundNo.	Fungi				Gram positive bacteria		Gram negative bacteria	
	*A. fumigatus*	*P. italicum*	*C. albicans*	*G. candidum*	*S. aureus*	*B. subtilis*	*P. aeruginosa*	*E. coli*
**4a**	125	125	N.A.	31.25	62.5	15.63	N.A.	500
**4c**	31.3	15.6	N.A.	3.9	1.95	0.12	N.A.	62.5
**7c**	31.3	15.6	N.A.	7.81	62.5	31.3	N.A.	500
**8b**	15.6	7.8	N.A.	0.12	1.95	0.06	N.A.	31.3
**8c**	125	62.5	N.A.	7.81	15.6	7.8	N.A.	500
**9a**	14.1	N.A.	N.A.	0.11	1.75	0.12	N.A.	26.5
**9b**	0.9	15.3	N.A.	0.08	1.95	8.5	N.A.	500
Amphotericin B	0.49	1.95	15.63	0.015	N.A.	N.A.	N.A.	N.A.
Ampicillin	N.A.	N.A.	N.A.	N.A.	0.02	0.007	N.A.	N.A.
Gentamicin	N.A.	N.A.	N.A.	N.A.	N.A.	N.A.	62.5	0.98

## 3. Experimental

### 3.1. General

Melting points were determined using an electrothermal Gallenkamp apparatus and are reported uncorrected. IR spectra were recorded in KBr using a Pye Unicam SP-1000 Spectrometer. ^1^H-NMR spectra were recorded using DMSO-d_6_ solutions on a Varian EM-300 MHz Spectrometer and chemical shifts are reported in ppm relative to that of TMS, which was used as an internal standard. Mass spectra were recorded using a AEI MS 30 mass spectrometer operating at 70 eV. Elemental analyses were carried out by using the Microanalytical Center of Cairo University, Giza, Egypt. The enaminones **1a**–**d** were prepared as previously reported [11]. ^13^C-NMR of compounds **7d**, **8d**, **9c** and **9d** could not be recorded due to the fact they precipitated in DMSO.

### 3.2. Reaction of Enaminones ***1a**–**d*** with Hydrazonoyl Chloride ***2***

To a stirred solution of the appropriate enaminones **1a**–**d** (2.5 mmol) and the hydrazonoyl chloride **2** (0.49 g, 2.5 mmol) in dry dioxane (30 mL), was added triethylamine (0.5 mL), and the mixture was heated for 5 h. The precipitated triethylamine hydrochloride was filtered off, the filtrate was concentrated under reduced pressure, and the residue was triturated with methanol. The solid product so formed in each case, was collected by filtration, washed with water, dried, and recrystallized from ethanol to afford the corresponding 1,3,4-thiadiazole derivatives **4**. The products **4a**–**d** prepared are listed below together with their physical constants.

*5-[3-Acetyl-1-phenyl-1H-pyrazole-4-carbonyl]-2-benzoylimino-3-(4-methoxyphenyl)-3H-[1,3,4]thiadiazole* (**4a**). Yellowish-red solid (81% yield), mp 160–162 °C; IR (KBr) ν_max_ 1685, 1642, 1609 (3C=O), 1554 (C=N) cm^−1^; ^1^H-NMR (DMSO-d_6_) δ 2.38 (s, 3H, COCH_3_), 3.86 (s, 3H, OCH_3_), 7.02–7.48 (m, 5H, Ar-H), 7.50–7.63 (m, 5H, Ar-H) 7.87 (d, *J* = 8 Hz, 2H, Ar-H), 8.11 (d, *J* = 8 Hz, 2H, Ar-H), 9.31 (s, 1H, pyrazolyl-H); ^13^C-NMR (DMSO-d_6_) δ: 25.32, 55.48, 106.16, 114.39, 115.55, 115.77, 120.31, 126.46, 127.34, 128.42, 129.63, 132.50, 146.20, 148.56, 155.81, 155.86, 160.29, 162.02, 176.20, 190.21, 206.60. MS *m/z* (%) 524 (M^+^+1, 2), 523 (M^+^, 2), 408 (7), 325 (2), 285 (2), 213 (10), 105 (24), 98 (100), 77 (27). Anal. Calcd. for C_28_H_21_N_5_O_4_S (523.56): C, 64.23; H, 4.04; N, 13.38. Found: C, 64.44; H, 4.17; N, 13.58%.

*5-[3-Acetyl-1-phenyl-1H-pyrazole-4-carbonyl]-2-benzoylimino-3-(4-methylphenyl)-3H-[1,3,4]thiadiazole* (**4b**). Yellowish-red solid (82% yield), mp > 300 °C; IR (KBr) ν_max_, 1642, 1617, 1610 (3C=O), 1559 (C=N) cm^−1^; ^1^H-NMR (DMSO-d_6_) δ 2.44 (s, 3H, COCH_3_), 2.49 (s, 3H, CH_3_-Ar), 7.44 (d, *J* = 8 Hz, 2H, Ar-H), 7.47–7.51 (m, 5H, Ar-H), 7.52 (d, *J* = 8 Hz, 2H, Ar-H), 7.61–8.11 (m, 5H, Ar-H), 9.32 (s, 1H, pyrazolyl-H); ^13^C-NMR (DMSO-d_6_) δ: 11.58, 21.53, 114.0, 116.35, 120.48, 124.46, 126.91, 129.32, 129.95, 131.40, 132.03, 133.19, 136.13, 137.38, 143.38, 144.17, 150.98, 160.34, 174.32, 189.95, 196.30. MS *m/z* (%) 508 (M^+^+1, 9), 507 (M^+^, 15), 392 (11), 213 (57), 121 (11), 105 (46), 98 (100), 77 (43). Anal. Calcd. for C_28_H_21_N_5_O_3_S (507.56): C, 66.26; H, 4.17; N, 13.80. Found: C, 66.39; H, 4.34; N, 13.94%.

*5-[3-Acetyl-1-phenyl-1H-pyrazole-4-carbonyl]-2-benzoylimino-3-(4-chlorophenyl)-3H-[1,3,4]thiadiazole* (**4c**). Yellow solid (80% yield), mp 158–160 °C; IR (KBr) ν_max_ 1689, 1638, 1610 (3C=O), 1546 (C=N) cm^−1^; ^1^H-NMR (DMSO-d_6_) δ 2.38 (s, 3H, COCH_3_), 7.63–7.69 (m, 5H, Ar-H), 7.89–8.15 (m, 5H, Ar-H) 7.43 (d, *J* = 8 Hz, 2H, Ar-H), 7.49 (d, *J* = 8 Hz, 2H, Ar-H), 9.34 (s, 1H, pyrazolyl-H); ^13^C-NMR (DMSO-d_6_) δ: 21.45, 112.64, 115.31, 119.72, 122.22, 126.15, 126.80, 127.78, 128.40, 128.81, 131.20, 132.58, 135.70, 138.47, 143.71, 151.17, 162.0, 176.06, 190.30, 206.03. MS *m/z* (%) 528 (M^+^+1, 2), 527 (M^+^, 8), 412 (4), 312 (36), 111(6), 105 (45), 98 (100), 77 (39). Anal. Calcd. for C_27_H_18_ClN_5_O_3_S (527.98): C, 61.42; H, 3.44; N, 13.26. Found: C, 61.62; H, 3.51; N, 13.45%.

*5-[3-Acetyl-1-phenyl-1H-pyrazole-4-carbonyl]-2-benzoylimino-3-(4-nitrophenyl)-3H-[1,3,4]thiadiazole* (**4d**). Brown solid (78% yield), mp 236–238 °C; IR (KBr) ν_max_ 1694, 1636, 1604 (3C=O), 1548 (C=N) cm^−1^; ^1^H-NMR (DMSO-d_6_) δ 2.40 (s, 3H, COCH_3_), 7.48–8.45 (m, 14H, Ar-H), 9.54 (s, 1H, pyrazole H); ^13^C-NMR (DMSO-d_6_) δ: 23.14, 113.24, 116.04, 119.56, 124.12, 126.26, 127.01, 127.71, 129.0, 129.21, 131.0, 132.69, 136.35, 138.17, 142.08, 152.11, 159.90, 172.14, 190.17, 201.37. MS *m/z* (%) 539 (M^+^+1, 8), 538 (M^+^, 4), 423 (6), 406 (10), 356 (5), 292 (5), 105 (52), 98 (100), 77 (28). Anal. Calcd. for C_27_H_18_N_6_O_5_S (538.54): C, 60.22; H, 3.37; N, 15.61. Found: C, 60.05; H, 3.28; N, 15.39%.

### 3.3. Reaction of Enaminones ***1a**–**d*** with Heterocyclic Amines

*General procedure*: To a solution of the appropriate enaminone **1a**–**d** (5 mmol) in acetic acid (20 mL) was added the appropriate heterocyclic amine (3-aminotriazole or 2-aminobenzimidazole 5 ,mmol). The mixture was stirred at reflux for 6 h then cooled. The formed solid was separated by filtration and recrystallized from dioxane to give compounds **7a**–**d** and **8a**–**d**, respectively.

*5-[2-Benzoylimino-3-(4-methoxyphenyl)-1,3,4-thiadiazol-5-yl]-[1,2,4]triazolo[1,5-a]pyrimidine* (**7a**). Yellow solid (80% yield), mp 235–236 °C; IR (KBr) ν_max_ 1604 (C=O), 1558 (C=N) cm^−1^; ^1^H-NMR (DMSO-d_6_) δ 3.88 (s, 3H, OCH_3_), 7.48–7.59 (m, 5H, Ar-H), 7.24 (d, *J* = 8 Hz, 2H, ArH), 7.97 (d, *J* = 8 Hz, 2H, Ar-H), 8.12 (d, 1H, *J* = 4.5 Hz, pyrimidinyl-H), 8.14 (d, 1H, *J* = 4.5 Hz, pyrimidinyl-H), 9.05 (s, 1H, triazolyl-H); ^13^C-NMR (DMSO-d_6_) δ: 53.48, 115.58, 121.65, 125.79, 126.29, 128.80, 129.23, 129.53, 130.52, 130.98, 131.19, 143.87, 144.99, 147.10, 151.72, 159.83, 175.22. MS *m/z* (%) 430 (M^+^+1, 6), 429 (M^+^, 40), 352 (3), 306 (26), 179 (2), 121 (43), 105 (100), 97 (8), 77 (66). Anal. Calcd. for C_21_H_15_N_7_O_2_S (429.46): C, 58.73; H, 3.52; N, 22.83. Found: C, 58.53; H, 3.38; N, 22.64%.

*5-[2-Benzoylimino-3-(4-methylphenyl)-1,3,4-thiadiazol-5-yl]-[1,2,4]triazolo[1,5-a]pyrimidine* (**7b**). Yellow solid (79% yield), mp > 300 °C; IR (KBr) ν_max_ 1602 (C=O), 1540 (C=N) cm^−1^; ^1^H-NMR (DMSO-d_6_) δ 2.46 (s, 3H, CH_3_), 7.50–7.61 (m, 5H, ArH), 8.13 (d, *J* = 8 Hz, 2H, ArH), 8.16 (d, *J* = 8 Hz, 2H, ArH), 7.95 (d, *J* = 4.5 Hz, 1H, pyrimidinyl-H), 8.97 (s, 1H, triazolyl-H), 9.05 (d, *J* = 4.5 Hz, 1H, pyrimidinyl-H); ^13^C-NMR (DMSO-d_6_) δ: 18.27, 114.01, 120.24, 123.27, 127.06, 128.18, 129.05, 130.47, 130.42, 132.48, 134.35, 142.54, 144.84, 148.21, 150.70, 158.16, 172.02. MS *m/z* (%) 414 (M^+^+1, 3), 413 (M^+^, 11), 306 (10), 105 (100), 98 (2), 77 (62). Anal. Calcd. for C_21_H_15_N_7_OS (413.46): C, 61.00; H, 3.66; N, 23.71. Found: C, 61.25; H, 3.47; N, 23.51%.

*5-[2-Benzoylimino-3-(4-chlorophenyl)-1,3,4-thiadiazol-5-yl]-[1,2,4]triazolo[1,5-a]pyrimidine* (**7c**). Pale brown solid (76% yield), mp > 300 °C; IR (KBr) ν_max_ 1610 (C=O), 1545 (C=N) cm^−1^; ^1^H-NMR (DMSO-d_6_) δ 7.38 (d, *J* = 8 Hz, 2H, ArH), 7.49–7.96 (m, 5H, ArH), 8.12 (d, *J* = 8 Hz, 2H, ArH), 8.24 (d, *J* = 4.5 Hz, 1H, pyrimidinyl-H), 8.45 (d, *J* = 4.5 Hz, 1H, pyrimidinyl-H), 8.61 (s, 1H, triazolyl-H); ^13^C-NMR (DMSO-d_6_) δ: 114.21, 115.16, 118.27, 120.03, 122.10, 124.24, 126.57, 128.60, 135.28, 137.67, 142.11, 143.95, 147.09, 150.79, 155.20, 171.11. MS *m/z* (%) 435 (M^+^+2, 3), 434 (M^+^+1, 4), 433 (M^+^, 9), 356 (3), 306 (10), 105 (100), 98 (66), 77 (80). Anal. Calcd. for C_20_H_12_ClN_7_OS (433.87): C, 55.36; H, 2.79; N, 22.60. Found: C, 55.26; H, 2.64; N, 22.49%.

*5-(2-Benzoylimino-3-(4-nitrophenyl)-1,3,4-thiadiazol-5-yl)-[1,2,4]triazolo[1,5-a]pyrimidine* (**7d**). Brown solid (79% yield), mp > 300 °C; IR (KBr) ν_max_ 1612 (C=O), 1540 (C=N) cm^−1^; ^1^H-NMR (DMSO-d_6_) δ 7.53–7.64 (m, 5H, ArH), 8.26 (d, *J* = 8 Hz, 2H, ArH), 8.52 (d, *J* = 8 Hz, 2H, ArH), 8.56 (d, *J* = 4.5 Hz, 1H, pyrimidinyl-H), 8.59 (s, 1H, triazolyl-H), 9.00 (d, *J* = 4.5 Hz, 1H, pyrimidinyl-H); ^13^C-NMR (DMSO-d_6_) δ: the sample precipitated. MS *m/z* (%) 444 (M^+^, 17), 415 (3), 367 (4), 306 (4), 299 (2), 121 (3), 105 (100), 90 (4), 77 (42). Anal. Calcd. for C_20_H_12_N_8_O_3_S (444.43): C, 54.05; H, 2.72; N, 25.21. Found: C, 54.18; H, 2.57; N, 25.05%.

*4-[2-Benzoylimino-3-(4-methoxyphenyl)-1,3,4-thiadiazol-5-yl]-benzimidazo[1,2-a]pyrimidine* (**8a**). Yellow solid (78% yield), mp 265–266 °C; IR (KBr) ν_max_ 1642 (C=O), 1604 (C=N) cm^−1^; ^1^H-NMR (DMSO-d_6_) δ 3.88 (s, 3H, OCH_3_), 7.17–7.59 (m, 4H, Ar-H), 7.85 (d, *J* = 7 Hz, 2H, Ar-H), 7.91–8.09 (m, 5H, Ar-H), 8.10 (d, *J* = 7 Hz, 2H, Ar-H), 8.14 (d, *J* = 4.5 Hz, 1H, pyrimidinyl-H), 9.68 (d, *J* = 4.5 Hz, 1H, pyrimidinyl-H); ^13^C-NMR (DMSO-d_6_) δ: 52.10, 112.70, 113.33, 115.37, 121.57, 121.77, 122.91, 125.85, 126.38, 126.52, 130.67, 133.72, 134.74, 135.08, 135.48, 137.24, 144.36, 148.74, 149.50, 150.80, 157.13, 169.26. MS *m/z* (%) 479 (M^+^+1, 27), 478 (M^+^, 29), 355 (25), 194 (12), 121(10), 105 (83), 77 (98). Anal. Calcd. for C_26_H_18_N_6_O_2_S (478.53): C, 65.26; H, 3.79; N, 17.56. Found: C, 65.08; H, 3.56; N, 17.33%.

*4-[2-Benzoylimino-3-(4-methylphenyl)-1,3,4-thiadiazol-5-yl]-benzimidazo[1,2-a]pyrimidine* (**8b**). Orange solid (73% yield), mp 270 °C; IR (KBr) ν_max_ 1610 (C=O), 1570 (C=N) cm^−1^; ^1^H-NMR (DMSO-d_6_) δ 2.35 (s, 3H, CH_3_), 6.89–7.35 (m, 4H, Ar-H), 7.45 (d, *J* = 8 Hz, 2H, Ar-H), 7.74–8.01 (m, 5H, Ar-H), 8.0 (d, *J* = 8 Hz, 2H, Ar-H), 8.08 (d, *J* = 4.5 Hz, 1H, pyrimidinyl-H), 9.24 (d, *J* = 4.5 Hz, 1H, pyrimidinyl-H); ^13^C-NMR (DMSO-d_6_) δ: 14.15, 115.63, 116.0, 121.22, 122.77, 125.49, 126.02, 128.01, 128.51, 128.59, 129.27, 129.82, 130.04, 131.50, 134.74, 136.68, 139.23, 140.82, 142.08, 152.37, 157.90, 168.95. MS *m/z* (%) 462 (M^+^, 18), 385 (9), 355 (7), 285 (2), 195 (23), 105 (100), 77 (53). Anal. Calcd. for C_26_H_18_N_6_OS (462.53): C, 67.52; H, 3.92; N, 18.17. Found: C, 67.40; H, 3.84; N, 18.27%.

*4-[2-Benzoylimino-3-(4-chlorophenyl)-1,3,4-thiadiazol-5-yl]-benzimidazo[1,2-a]pyrimidine* (**8c**). Dark-orange solid (74% yield), mp > 300 °C; IR (KBr) ν_max_ 1635 (C=O), 1556 (C=N) cm^−1^; ^1^H-NMR (DMSO-d_6_) δ 7.47–7.64 (m, 4H, ArH), 7.85–8.15 (m, 5H, ArH), 7.71–7.80 (m, 4H, ArH), 8.37 (d, *J* = 4.5 Hz, 1H, pyrimidinyl-H), 9.71 (d, *J* = 4.5 Hz, 1H, pyrimidinyl-H); ^13^C-NMR (DMSO-d_6_) δ: 111.0, 114.68, 117.21, 123.72, 124.26, 127.23, 128.83, 130.55, 134.84, 135.41, 136.09, 137.50, 139.26, 145.20, 145.54, 147.70, 148.08, 149.24, 158.71, 159.67, 169.62. MS *m/z* (%) 484 (M^+^+2, 13), 482 (M^+^, 32), 397 (10), 395 (8), 194 (22), 127 (18), 105 (100), 90 (49), 77 (71). Anal. Calcd. for C_25_H_15_ClN_6_OS (482.95): C, 62.17; H, 3.13; N, 17.40. Found: C, 62.30; H, 3.06; N, 17.30%.

4-[2-Benzoylimino-3-(4-nitrophenyl)-1,3,4-thiadiazol-5-yl]-benzimidazo[1,2-a]pyrimidine (**8d**). Brown solid (82% yield), mp > 300 °C; IR (KBr) ν_max_ 1625 (C=O), 1525 (C=N) cm^−1^; ^1^H-NMR (DMSO-d_6_) δ 7.51–7.61(m, 5H, ArH), 7.86–7.95 (m, 4H, Ar-H), 8.15 (d, *J* = 5 Hz, 1H, pyrimidinyl-H) 8.36–8.54 (m, 4H, ArH), 9.69 (d, *J* = 5 Hz, 1H, pyrimidinyl-H); ^13^C-NMR (DMSO-d_6_) δ: the sample precipitated. MS *m/z* (%) 493 (M^+^, 1), 340 (2), 122 (2), 105 (45), 98 (100), 77 (44), 55 (11). Anal. Calcd. for C_25_H_15_N_7_O_3_S (493.50): C, 60.84; H, 3.06; N, 19.87. Found: C, 60.65; H, 3.21; N, 19.70%.

### 3.4. Reaction of Enaminones ***1a**–**d*** with Hydrazine Hydrate

A mixture of the appropriate enaminones **1a**–d (5 mmol) and hydrazine hydrate (5 mL) in absolute ethanol was stirred at reflux for 10 h and cooled. The solid formed was separated by filtration and recrystallized from ethanol/dioxane mixture to give **9a**–**d**.

*N-[3-(4-Methoxyphenyl)-5-(1H-pyrazol-3-yl)-3H-[1,3,4]thiadiazol-2-ylidene]-benzamide* (**9a**). White solid (83% yield), mp 200–202 °C; IR (KBr) ν_max_ 3297 (NH), 1611 (C=O), 1549 (C=N) cm^−1^; ^1^H-NMR (DMSO-d_6_) δ 3.69 (s, 3H, OCH_3_), 7.08–7.11 (m, 9H, Ar-H), 7.88 (d, *J* = 8 Hz, 1H, pyrazolyl-H), 7.95 (d, *J* = 8 Hz, 1H, pyrazolyl-H), 9.0 (s, 1H, NH); ^13^C-NMR (DMSO-d_6_) δ: 56.12, 114.31, 116.09, 119.11, 121.64, 125.32, 125.32, 128.08, 128.47, 129.10, 136.20, 142.62, 154.03, 160.12, 170.13. MS *m/z* (%) 377 (M^+^, 2), 333 (1), 288 (38), 266 (100), 251 (66), 179 (3), 149 (8), 133 (12), 121 (19), 104 (43), 98 (2), 77 (34). Anal. Calcd. for C_19_H_15_N_5_O_2_S (377.42): C, 60.46; H, 4.01; N, 18.56. Found: C, 60.49; H, 4.11; N, 18.29%.

*N-[3-(4-Methylphenyl)-5-(1H-pyrazol-3-yl)-3H-[1,3,4]thiadiazol-2-ylidene]-benzamide* (**9b**). White solid, (86% yield), mp 220–222 °C; IR (KBr) ν_max_ 3300 (NH), 1609 (C=O), 1545 (C=N) cm^−1^; ^1^H-NMR (DMSO-d_6_) δ 2.38 (s, 3H, CH_3_), 7.04–7.06 (m, 5H, Ar-H), 7.34–7.37 (m, 4H, Ar-H) 7.46 (d, *J* = 8 Hz, 1H, pyrazolyl-H), 7.88 (d, *J* = 8 Hz, 1H, pyrazolyl-H), 9.15 (s, 1H, NH); ^13^C-NMR (DMSO-d_6_) δ: 15.62, 114.28, 117.11, 120.0, 121.45, 124.13, 126.27, 128.99, 129.27, 130.58, 135.24, 144.17, 152.07, 158.08, 171.22. MS *m/z* (%) 361 (M^+^, 2), 272 (3), 250 (61), 225 (4), 146 (5), 104 (24), 91 (20), 77 (16), 65 (14), 46 (100). Anal. Calcd. for C_19_H_15_N_5_OS (361.42): C, 63.14; H, 4.18; N, 19.38. Found: C, 63.0; H, 4.08; N, 19.09%.

*N-[3-(4-Chlorophenyl)-5-(1H-pyrazol-3-yl)-3H-[1,3,4]thiadiazol-2-ylidene]-benzamide* (**9c**). Pale yellow solid (78% yield), mp 278–280 °C; IR (KBr) ν_max_ 3315 (NH), 1607(C=O), 1546 (C=N) cm^−1^; ^1^H-NMR (DMSO-d_6_) δ 7.05–7.67 (m, 9H, Ar-H), 7.74 (d, *J* = 8 Hz, 1H, pyrazolyl-H), 8.28 (d, *J* = 8 Hz, 1H, pyrazolyl-H), 10.22 (s, 1H, NH); ^13^C-NMR (DMSO-d_6_) δ: the sample precipitated. MS *m/z* (%) 383 (M^+^+2, 2), 382 (M^+^1, 1), 381(M^+^, 5), 272 (22), 271 (20), 270 (58), 192 (21), 105 (21), 111(52), 77 (100). Anal. Calcd. for C_18_H_12_ClN_5_OS (381.84): C, 56.62; H, 3.17; N, 18.34. Found: C, 56.48; H, 3.04; N, 18.25%.

*N-[3-(4-Nitrophenyl)-5-(1H-pyrazol-3-yl)-3H-[1,3,4]thiadiazol-2-ylidene]-benzamide* (**9d**). Brown solid (81% yield), mp 280–282 °C; IR (KBr) ν_max_ 3302 (NH), 1605 (C=O), 1551 (C=N) cm^−1^; ^1^H-NMR (DMSO-d_6_) δ 7.51–7.58 (m, 5H, Ar-H), 7.73–7.77 (m, 4H, Ar-H) 7.86 (d, *J* = 8 Hz, 1H, pyrazolyl-H), 8.19 (d, *J* = 8 Hz, 1H, pyrazolyl-H), 10.34 (s, 1H, NH); ^13^C-NMR (DMSO-d_6_) δ: the sample precipitated. MS *m/z* (%) 392 (M^+^, 20), 298 (7), 297 (5), 295 (5), 281 (100), 251 (67), 235 (11), 149 (21), 132 (44), 118 (24), 104 (78), 90 (25), 77 (71). Anal. Calcd. for C_18_H_12_N_6_O_3_S (392.39): C, 55.10; H, 3.08; N, 21.42. Found: C, 55.0; H, 3.21; N, 21.20%.

### 3.5. Microbiological Studies

#### 3.5.1. Agar Diffusion Well Method to Determine the Antimicrobial Activity

The microorganism inoculums were uniformly spread using sterile cotton swabs on a sterile Petri dish containing malt extract agar (for fungi) and nutrient agar (for bacteria). Each sample (100 μL) was added to each well (6 mm diameter holes cut in the agar gel, 20 mm apart from one another). The systems were incubated for 24–48 h at 37 °C (for bacteria) and at 28 °C (for fungi). After incubation, microorganism growth was observed. Inhibition of the bacterial and fungal growth were measured in mm. Tests were performed in triplicate [22].

#### 3.5.2. Minimal Inhibitory Concentration (MIC) Measurement

The bacteriostatic activity of the active compounds (having inhibition zones (IZ) ≥ 16 mm) was then evaluated using the two fold serial dilution technique. Two fold serial dilutions of the tested compounds solutions were prepared using the proper nutrient broth. The final concentration of the solutions was 132; 66; 33; 16.5; and 8.25 mg/mL. The tubes were then inoculated with the test organisms, grown in their suitable broth at 37 °C for 24 h for bacteria (about 1 × 10^8^ CFU/mL), each 5 mL received 0.1 mL of the above inoculum and incubated at 37 °C for 24 h. The lowest concentration showing no growth was taken as the minimum inhibitory concentration (MIC).

## 4. Conclusions

New series of 1,3,4-thiadiazoles incorporating pyrazole, triazolopyrimidine and benzimidazo-pyrimidines were synthesized *via* reaction of 1,3,4-thiadiazolenaminones with hydrazonoyl chloride and nitrogen nucleophiles. The structure of the new products was established based on elemental and spectral analysis. The antimicrobial activity results of the products indicated that some of the newly synthesized compounds showed promising activity. 
